# Glyco-iELISA: a highly sensitive and unambiguous serological method to diagnose STEC-HUS caused by serotype O157

**DOI:** 10.1007/s00467-018-4118-9

**Published:** 2018-10-26

**Authors:** Kioa L. Wijnsma, Susan T. Veissi, Sheila A. M. van Bommel, Rik Heuver, Elena B. Volokhina, Diego J. Comerci, Juan E. Ugalde, Nicole C. A. J. van de Kar, Lambertus P. W. J. van den Heuvel

**Affiliations:** 1 Radboud Institute for Molecular Life Sciences, Amalia Children’s Hospital, Department of Pediatric Nephrology, Radboud University Medical Center, P.O. Box 9101, 6500 HB Nijmegen, The Netherlands; 2 Department of Laboratory Medicine, Radboud University Medical Center, Nijmegen, The Netherlands; 3 Instituto de Investigaciones Biotecnológicas Dr. Rodolfo A. Ugalde, Universidad Nacional de San Martín, Buenos Aires, Argentina; 4 Department of Development and Regeneration, University Hospital Leuven, Leuven, Belgium

**Keywords:** Shiga toxin–producing *E. coli*, Hemolytic uremic syndrome, Serology, Glycoproteins

## Abstract

**Background:**

Providing proof of presence of Shiga toxin–producing *E. coli* (STEC) infection forms the basis for differentiating STEC-hemolytic uremic syndrome (HUS) and atypical HUS. As the gold standard to diagnose STEC-HUS has limitations, using ELISA to detect serum antibodies against STEC lipopolysaccharides (LPS) has proven additional value. Yet, conventional LPS-ELISA has drawbacks, most importantly presence of cross-reactivity due to the conserved lipid A part of LPS. The newly described glyco-iELISA tackles this issue by using modified LPS that eliminates the lipid A part. Here, the incremental value of glyco-iELISA compared to LPS-ELISA is assessed.

**Methods:**

A retrospective study was performed including all pediatric patients (*n* = 51) presenting with a clinical pattern of STEC-HUS between 1990 and 2014 in our hospital. Subsequently, the diagnostic value of glyco-iELISA was evaluated in a retrospective nationwide study (*n* = 264) of patients with thrombotic microangiopathy (TMA). LPS- and glyco-iELISA were performed to detect IgM against STEC serotype O157. Both serological tests were compared with each other and with fecal diagnostics.

**Results:**

Glyco-iELISA is highly sensitive and has no cross-reactivity. In the single-center cohort, fecal diagnostics, LPS-ELISA, and glyco-iELISA identified STEC O157 infection in 43%, 65%, and 78% of patients, respectively. Combining glyco-iELISA with fecal diagnostics, STEC infection due to O157 was detected in 89% of patients. In the nationwide cohort, 19 additional patients (8%) were diagnosed with STEC-HUS by glyco-iELISA.

**Conclusion:**

This study shows that using glyco-iELISA to detect IgM against STEC serotype O157 has clear benefit compared to conventional LPS-ELISA, contributing to optimal diagnostics in STEC-HUS.

## Introduction

Providing proof of the presence of Shiga toxin (Stx)–producing *Escherichia coli* (STEC) infection forms the basis for differentiation between STEC-hemolytic uremic syndrome (HUS) and atypical HUS (aHUS). Both are important causes to bear in mind in patients who present with signs indicative of thrombotic microangiopathy (TMA), hemolytic anemia, thrombocytopenia, and acute kidney injury. The current gold standard to detect STEC infection involves fecal examination by culture and detection of *Stx-*encoding genes by polymerase chain reaction (PCR) [1–3]. However, detection of STEC in the feces is limited due to the natural course of the disease. Furthermore, due to low inoculums, detection of STEC in the feces becomes increasingly difficult as the disease progresses. Despite new and upcoming fecal diagnostic techniques, such as molecular serotyping, sole use of fecal diagnostics is not yet sufficient in establishing STEC infection [4].

As the gold standard diagnostic for STEC-HUS has various limitations, the use of enzyme-linked immunosorbent assay (ELISA) to detect serum antibodies against the lipopolysaccharides (LPS; LPS-ELISA) of STEC has proven its value [2, 5, 6]. As previously shown, the combination of fecal diagnostics with LPS-ELISA to detect immunoglobulin M (IgM) against STEC serotype O157 clearly has an added value in the detection of STEC infection as the cause of HUS [6]. Yet, the conventional LPS-ELISA, in which plates are coated with purified LPS, has various drawbacks. The most important limitation is the presence of cross-reactivity caused by the conserved lipid A part of the LPS molecule [5, 7, 8]. The LPS structure consists of a lipid A part, an outer and inner core, and an O-antigen, of which the latter has the highest immunogenic activity. Since the lipid A is also present in other strains of STEC, and even other gram-negative bacteria, this could lead to cross-reactivity and ultimately lead to false-positive results [7, 9, 10]. Although O157 antigen remains the most prevalent serotype associated with STEC-HUS, other non-O157 strains (such as O26, O103, O104, O111, O55) are increasingly detected. Differentiation between the different STEC serotypes is important considering the variation in clinical presentation, course of the disease per serotype, and the epidemiological consequences [2, 6, 11].

To tackle the aforementioned issues, the group of J.E. Ugalde and D.J. Comerci exploited a new ELISA technique—indirect glycoprotein-based ELISA (glyco-iELISA)—for the detection of STEC infection in HUS patients [8, 11]. This glyco-iELISA takes advantage of a bacterial glycoengineering technology to develop and produce recombinant serotype-specific glycoproteins consisting of the O157 polysaccharide attached to the protein carrier acceptor AcrA (O157-Acr), in the absence of the lipid A part. As shown by Melli et al., the glyco-iELISA was able to diagnose STEC in HUS patients, even in cases where fecal diagnostics failed [11]. More importantly, due to the absence of the lipid A structure of LPS in the glycoprotein constructs used in this assay, potential cross-reactivity is counteracted. Thus, glyco-iELISA appears to be a highly sensitive and specific assay [8].

Up until now, differentiation between STEC-HUS and aHUS remains a clinical conundrum, which can be tackled with the introduction of the glyco-iELISA [12]. Although STEC-HUS and aHUS require a completely different treatment approach, and clinical outcome is divergent, discrimination in the acute phase remains challenging [13, 14]. Whereas diarrhea is the clinical hallmark of STEC-HUS, in up to 30% of the patients with aHUS, a gastrointestinal infection has been found [13, 14]. Moreover, aHUS is treated with one of the world’s most expensive orphan drugs: the humanized monoclonal antibody, eculizumab [15]. In comparison, the treatment of STEC-HUS is merely symptomatic and eculizumab is not indicated for the treatment of STEC-HUS [13, 14]. This underlines the importance of discriminating between these types of HUS and limiting the unnecessary use of eculizumab in STEC-HUS.

With this retrospective study, the clinical utility of the glyco-iELISA compared to LPS-ELISA is accessed in two cohorts. The first cohort comprises pediatric patients of a single center with strong clinical suspicion of STEC-HUS, of which all clinical data were gathered. The second cohort is nationwide cohort of patients with signs indicative of TMA of which STEC infection could be a potential cause.

## Methods

A retrospective single-center pilot study was performed, which included all pediatric patients who presented with a clinical pattern of STEC-HUS between 1990 and 2014 to the Pediatric Nephrology department of the Radboud University Medical Center (Radboudumc) Amalia Children’s Hospital. A clinical pattern of STEC-HUS was defined as signs of a TMA, together with (bloody) diarrhea or a family member with diarrhea. Signs of TMA were classified as signs indicative of hemolysis (low hemoglobin, elevated LDH, depleted haptoglobin), acute renal injury, and thrombocytopenia of < 150 × 10^9^/l. Fever at presentation was defined as body temperature above 38.2 °C as reported by patients and/or parents. Anuria was defined as a urine production below 0.1 ml/kg/h for at least 12 h. The estimated glomerular filtration rate (eGFR) was calculated with the Schwartz formula (*k* 36.5) [16]. According to the pediatric Risk, Injury, Failure, Loss, End-stage renal disease (pRIFLE) criteria, renal injury is defined as an increased creatinine ×2 or decreased eGFR > 50%. Renal failure was defined as an increased creatinine ×3 or decreased eGFR > 75% [17]. The first day of illness was defined as the first day of diarrhea reported by the patient and/or parents. All available clinical and diagnostic data of these patients were collected in the STEC-HUS registry—an online web-based database. Residual material (serum) received during standard care was used to detect IgM antibodies against serotype O157 with LPS-ELISA as well as glyco-iELISA. This single-center study comprised the same patient cohort as previously described by Wijnsma et al. [6].

Subsequently, the diagnostic value of glyco-iELISA in STEC-HUS diagnosis was evaluated in a retrospective nationwide study. Since Radboudumc Amalia Children’s Hospital is the center of expertise for HUS patients in the Netherlands, LPS-ELISA for serotype O157 is only performed at the Translational Metabolic Laboratory in Radboudumc. This study included all residual sera samples from both pediatric and adult patients with signs of TMA. Of note, TMA was defined as thrombocytopenia, hemolytic anemia, and organ damage; yet, the underlying disease leading to the development of TMA was unknown at the time of sampling. Samples from these patients were sent to Radboudumc Amalia Children’s Hospital, between 2007 and 2014, from other university medical centers throughout the Netherlands for LPS-ELISA to diagnose or exclude STEC-HUS. All these residual sera samples were additionally tested for the presence of IgM antibodies against STEC O157 with glyco-iELISA. In addition, serum of 19 healthy adult controls was collected to determine the specificity of the assays and determine cutoff values.

This study does not fall within the remit of the Medical Research Involving Human Subjects Act (WMO). The study has been reviewed by the ethics committee on the basis of the Dutch Code of conduct for health research, the Dutch Code of conduct for responsible use, the Dutch Personal Data Protection Act and the Medical Treatment Agreement Act. The ethics committee has passed a positive judgment on the study (2017-3490).

### Index test: glyco-iELISA

The glyco-iELISA was performed as described by Melli et al. [11]. In brief, a microtiter plate was coated with recombinant glycoproteins (O157-AcrA) and incubated overnight at 4 °C. The following day, the plate was blocked with PBS-0.1% Tween 20 (PBST) + 0.5% skimmed milk for 1 h at room temperature (RT). Subsequently, diluted human serum samples (dilution of 1:800) were added and incubated for 1 h at RT. Next, the plate was washed and goat anti-human IgM (HRP-conjugated) antibody added and incubated for 1 h at RT. Hereafter, 3,3′,5,5′-tetramethylbenzidine (TMB, Sigma Aldrich) reagent as a substrate for HRP was added. Finally, the enzymatic reaction was stopped with 0.16 M sulfuric acid (H_2_SO_4_) and the absorbance measured at 450 nm (nm) with a spectrometer-based microtiter plate reader. Samples were considered positive when an optical density (OD) above 0.5 was observed.

Cross-reactivity was tested by adding sera from predetermined positive patients with various STEC serotype infections (resp. O26, O55, O103, O111, O145) together with predetermined negative patients, to an ELISA plate coated with glycoprotein serotype O157.

To study the effect of multiple freeze-thaw cycles on the patient blood samples, we freeze-thawed different previously determined positive samples, taken from three STEC-HUS patients, on 5 subsequent days. This resulted in samples of 1 up to 5 freeze-thaw cycles for each patient.

### Reference standard: fecal diagnostics and LPS-ELISA

Feces and serum from suspected STEC-HUS patients were collected as soon as possible after admission to the hospital. In cases where feces could not be obtained, a rectal swab was done. Serum was received from all patients during standard care and stored at − 80 °C until analysis. Fecal diagnostics and LPS-ELISA were performed as previously described [6]. Fecal diagnostics were considered positive when either PCR for Stx 1 and/or 2, the presence of fecal free Stx by using the verocell assay, or the fecal culture (using Sorbitol MacConkey agar plate) was positive for STEC. In the case of dubious test results, we considered the result as negative. After 2007, it became possible to send the STEC strains, isolated from the feces, to the Dutch National Institute for Public Health and Environment (RIVM) for further determination of the serotype, both O157 and non-O157. For LPS-ELISA, patients were considered positive when an optical density (OD) above 0.8 was observed. This cutoff was previously determined in close collaboration with Chart et al. [18].

### Assay characteristics

The response of both LPS-ELISA as glyco-iELISA was established by determining the lowest signal to detect a positive sample. In each setup of the plate, one previously determined positive and negative patient samples for the presence of antibodies against O157 antigens were diluted to different concentrations. Positive/negative ratios (P/N ratios) were calculated by dividing the OD obtained for each specific concentration. Subsequently, the coating antigen concentration was also taken into account when the sensitivity of the assays was compared.

### Statistics

For each assay, the mean, standard deviation (SD), and coefficient of variation (CV) were calculated. Furthermore, for the glyco-iELISA assays with patient screening, cutoff values were established using the following formula: mean control sera OD ± 2 SD.

Clinical values were expressed as valid percentages for categorical variables, and as the mean and SD, or median and 25–75% interquartile range (IQR) for continuous variables, as appropriate. The chi-square test was performed to compare categorical data. *p* values of < 0.05 were considered statistically significant. All graphs were created using GraphPad Prism software, version 5. For statistical analyses, SPSS software (version 22.0) was used.

## Results

### Patient characteristics

As previously published, during the period between 1990 and 2014, 65 patients with a clinical pattern of STEC-HUS presented in the Pediatric Nephrology Department of Radboudumc Amalia Children’s Hospital. Unfortunately, of the 65 patients, 14 patients had to be excluded from this analysis due the absence of residual material to test glyco-iELISA. The patient characteristics of the 51 STEC-HUS patients are described in Table 1. One patient with a highly severe presentation of STEC-HUS died due to a systemic inflammatory response syndrome. STEC infection was proven with both fecal diagnostics and serology.

**Table 1:** Characteristics of pediatric patients with STEC-HUS in a single-center cohort

Parameter	All patients (*n* = 51)
Male	47%
Age of onset in months	36 (23–65)
Symptoms at presentation	
Fever^a^	22%
Diarrhea, total^b^	96%
Of which bloody	78%
Anuria (defined as < 0.1 ml/kg/h)	57%
Blood pressure	
< p95	44%
> p95	56%
Neurological involvement	12% (*n* = 6)
Convulsions	8% (*n* = 4)
Coma	0%
Miscellaneous^c^	10% (*n* = 5)
Pancreas involvement	4%
Biochemical evaluation at presentation (reference range)	
Hemoglobin (mmol/l) (6.0–9.0)	5.3 (4.0–6.3)
White blood cells (×10^9^/l) (5.0–13.0)	14.5 (10.97–22.5)
Platelet count (×10^9^/l) (210–430)	45 (32–76)
Haptoglobin (g/l) (0.3–1.6)	< 0.08 (0.04–0.10)
LDH (U/l) *(<250)*	3929 (2525–5817)
Creatinine (μmol/l) (strongly depending age and body mass)	307 (190.5–430)
eGFR (ml/min.1.73m^2^)^d^ (> 90)	13 (8–23)
Treatment	
Dialysis	65%
Duration of dialysis in days	10 (7–14)
Erythrocytes transfusion	92%
≥ 3 transfusions	20%

Categorical values are expressed as percentage of total, and for continuous variables, the median with interquartile range (IQR) is expressed. Neurological involvement included areflexia, coma, epilepsy, and signs indicative of encephalopathy (decreased consciousness, abnormal behavior, amnesia, disorientation for time/person/place, disturbed speak, apraxia, hyperreflexia). P95: percentile for age and height [19]

^a^Fever, defined as body temperature above 38.2 °C, was reported by patients and/or parents

^b^Of note, we report two patients suspected of STEC-HUS without diarrhea. In one patient, STEC infection could be established by both fecal diagnostics (with rectal swab) as well as serology. In the second patient, serology for STEC O157 was negative and PCR was repeatedly reported as dubious. STEC-HUS seemed very likely, also in the light of good clinical recovery with minimal sequelae (mild proteinuria) and no recurrence after 6 years

^c^Either in combination with convulsion or as solo presentation. Other neurological symptoms reported were decreased consciousness (*n* = 3), ataxia (*n* = 1), and apathy (*n* = 1)

^d^All patients had signs indicative of renal injury according to the pRIFLE criteria. In total, 46 patients had renal failure based on the pRIFLE criteria

During the period between 1990 and 2017, a total of 264 serum samples from patients with acute TMA were sent to the laboratory of the HUS center of expertise at the Radboudumc Amalia Children’s Hospital. Of the 264 serum samples of this nationwide cohort, 212 samples were collected from 206 patients with TMA and 52 samples from 50 relatives of these patients. The median (range) age of the patients was 6 (0–73) years. The majority of the relatives were parents (*n* = 42), 5 siblings were tested, and 3 grandparents. Unfortunately, no clinical data were available from these patients as the samples were obtained from different hospitals in the Netherlands.

### Assay characteristics of glyco-iELISA

Since some samples were stored for quite some years, we accessed the stability of antibodies in our samples. However, up to 5 freeze-thaw cycles did not seem to have an effect on the OD values determined by glyco-iELISA (data not shown). Furthermore, in contrary to LPS-ELISA, glyco-iELISA shows low intra- and inter-assay variation (CV < 20%).

**Fig. 1:**
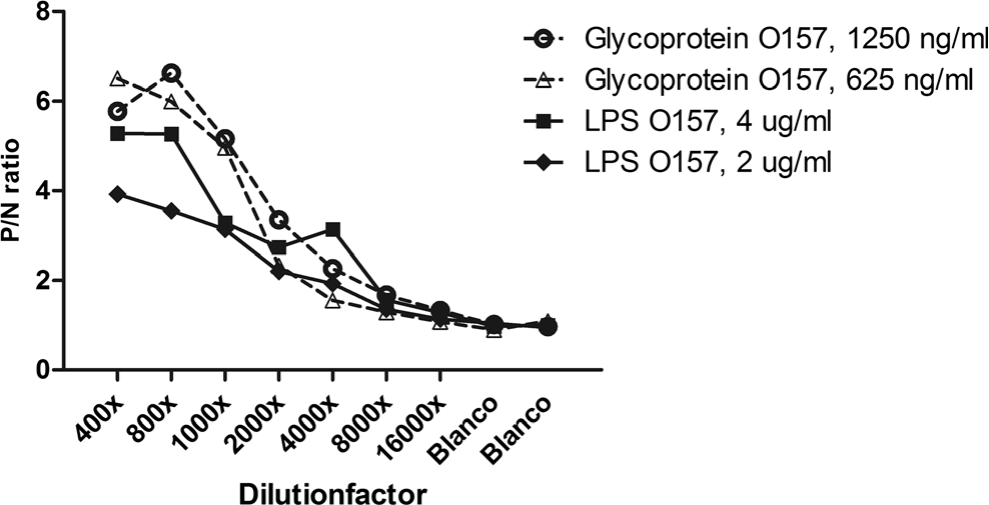
Response of LPS-ELISA versus glyco-iELISA. The response of both assays was assessed by determining the lowest signal at which a positive sample could still be detected for both LPS-ELISA as glyco-iELISA for STEC serotype O157. One previously determined positive and negative samples were each diluted with different concentrations. Positive/negative ratios were calculated by dividing the optical density obtained for each specific concentration. Subsequently, the coating antigen concentration was also taken into account when the accuracy of the assays was compared. LPS lipopolysaccharide, P/N positive negative ratio

**Fig. 2:**
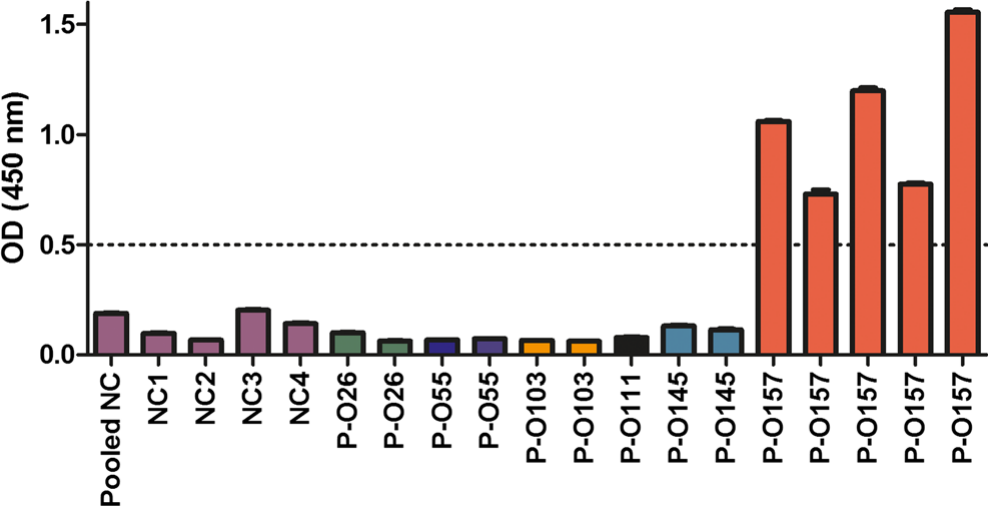
No cross-reactivity was observed with the glyco-iELISA for different STEC serotypes. No cross-reactivity with different STEC serotypes was observed with the O157 glyco-iELISA. After coating with glycoprotein O157, pooled sera of various healthy negative controls (NC) and 4 separate NC, together with sera of patients (P) with predetermined STEC infection with respective STEC serotypes O26, O55, O103, O111, O145, and O157 were added. Every bar represents one patient with STEC-HUS due to the serotype as indicated. The dotted bar represents the cutoff value of 0.5 optimal density (OD). Only the patients with STEC-HUS with serotype O157 were determined as positive, indicating no cross-reactivity

The discrimination capacity to detect positive and negative signals of LPS-ELISA and glyco-iELISA was determined for serological antibodies against O157 antigen and compared to each other (Fig. 1). Higher positive/negative (P/N) ratios were obtained with the glyco-iELISA, especially when using 1250 ng/ml glycoprotein with 800 times serum dilution (P/N ratio of 6.6) compared to LPS-ELISA. Furthermore, no cross-reactivity was observed for STEC serotypes O26, O111, O145, O103, and O55 antigens in the glyco-iELISA (Fig. 2).

### Sensitivity of glyco-iELISA

In total, 51 STEC-HUS patients with serology and clinical data were available and included in the analysis. Since the PCR for *Stx* genes was introduced in our hospital in 2011, it was only performed in 12 of the patients. In total, 9 patients had a proven STEC infection based on PCR for *Stx*, of which 7 were also confirmed with glyco-iELISA O157. The remaining two patients had a proven STEC infection with serotypes O55 and O26, explaining the negative glyco-iELISA result (see Table 2).

**Table 2:** Comparison between fecal diagnostics and glyco-iELISA for antibodies against STEC O157 antigen in patients with STEC-HUS in single-center cohort

Assays	Positive glyco-iELISA O157	Negative glyco-ELISA O157	Total number of patients
Positive fecal diagnostics^*^	18	4	22
Feces culture	*15*	*2*	*17*
Free fecal toxin (verocell assay)	*8*	*3*	*11*
PCR	*7*	*2*	*9*
Negative fecal diagnostics	22	7	29
Feces culture	*9*	*8*	*17*
Free fecal toxin (verocell assay)	*11*	*5*	*16*
PCR	*2*	*1*	*3*
Total number of patients	40	11	51

*Patients can be positive for each fecal diagnostic assay separate as well as all combined

*PCR* polymerase chain reaction

Furthermore, in 17 patients, further determination of STEC serotype took place based on fecal diagnostics and yielded 14 STEC infections with isolated strain serotype O157. All these patients also had serology for O157 tested with glyco-iELISA. This compared to serology tested by LPS-iELISA in which 2 patients tested negative.

### Time window to perform glyco-iELISA

**Fig. 3:**
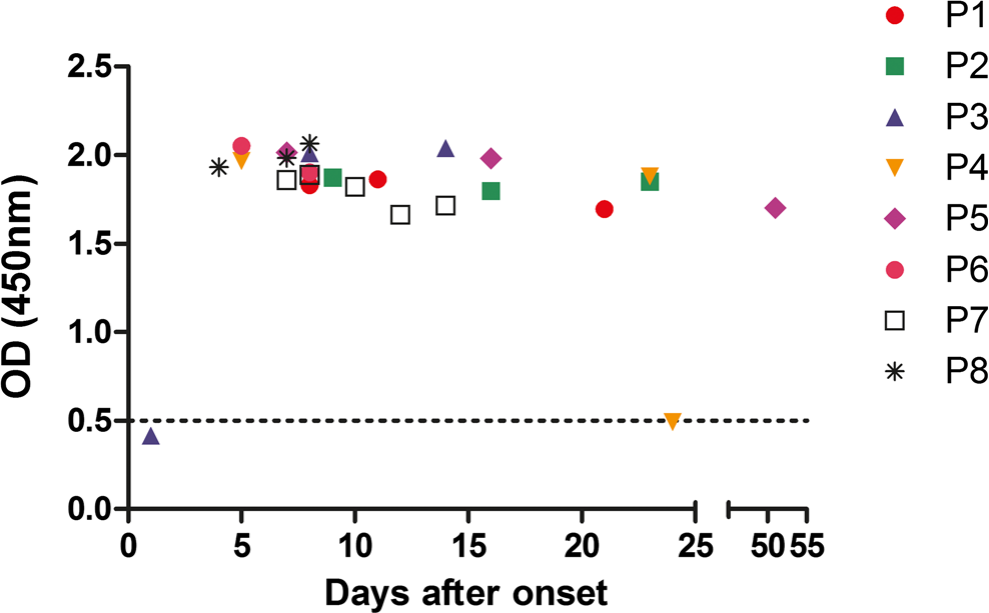
Time window to perform glyco-iELISA to detect IgM against STEC O157. From 8 STEC-HUS patients, multiple serum samples collected after the onset of diarrhea on different days during the course of their disease were tested using glyco-iELISA for the presence of antibodies against serotype O157. The dotted line represents the cutoff value of 0.5 optical density (OD) after which samples are categorized as positive for antibodies against serotype O157 in the glyco-iELISA

In 8 patients of our single-center cohort, serum was collected at various time points after the onset of diarrhea. These samples were used to examine the production of serum IgM antibodies (tested with glyco-iELISA) during the disease course of STEC-HUS (Fig. 3). A clear positive result for STEC O157 was already observed 1 day after the start of diarrhea. However, there is a strong variation between individuals. Patient 3 had no antibodies against STEC O157 on day 1; however, when tested on day 8 and 14, antibodies could be detected. Patient 4 (with negative serology on day 1) had a positive antibody response up to 23 days, after which the signal dropped significantly. Patient 5 had persisting antibodies until 51 days after the start of diarrhea.

### Single-center cohort of STEC-HUS patients

**Fig. 4:**
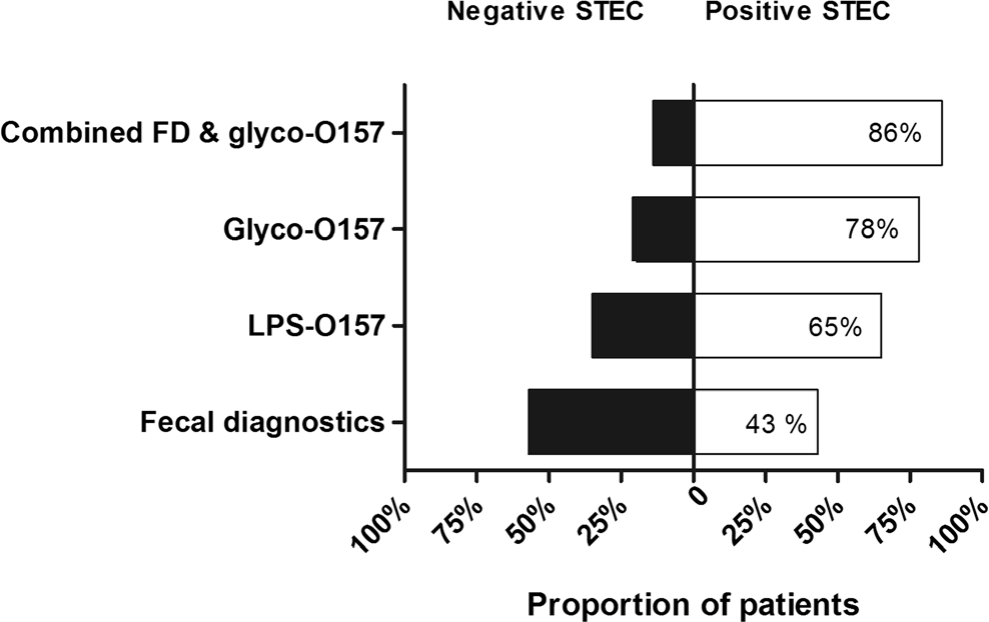
Proportion of pediatric HUS patients from single-center cohort tested positive for STEC infection with different diagnostic tools. The proportion of STEC-HUS patients in the single-center cohort per diagnostic test is described, given in percentages of the total 51 patients. The proportion of respectively positive (white bar) and negative patients (black bar) are depicted for fecal diagnostics (STEC detection by stool culture, free fecal Shiga toxin by verocell assay, PCR for Shiga toxin genes), LPS-ELISA, and glyco-iELISA for STEC serotype O157 (respectively LPS-O157 and Glyco-O157) and combined. When glyco-iELISA was combined with fecal diagnostics, the percentage of positive STEC patients increased up to 86%. fecal diagnostics, lipopolysaccharide, *STEC Shiga* toxin–producing *Escherichia coli*

Of the 51 STEC-HUS patients, 22 (43%) had a positive fecal diagnosis for STEC, 33 (65%) had positive serology based on LPS-ELISA against O157, and 40 (78%) had positive serology based on glyco-iELISA (see Table 3 and Fig. 4). In total, 3 patients appeared positive with O157 LPS-ELISA, whereas they were negative with glyco-iELISA. In 2 of these patients, fecal diagnostics revealed STEC infection with serotype O26 and O55, which explains the negative glyco-iELISA result, indicating a false-positive result (due to cross-reactivity) in the LPS-ELISA. In the third patient, the STEC strain was not further determined. Overall, the glyco-iELISA yielded 10 patients that were previously negative with LPS-ELISA. In conclusion, glyco-iELISA yielded significantly more patients positive for the presence of STEC when compared to fecal diagnostics (*p* < 0.0001) and LPS-ELISA (*p* = 0.04). Moreover, when combining fecal diagnostics with glyco-iELISA, STEC was detected in significantly more patients (86%, *p* = 0.03) than when combined with LPS-ELISA (73%).

**Table 3:** Comparison between LPS-ELISA and glyco-iELISA for antibodies against STEC O157 antigen in single-center cohort

Assays	Positive glyco-iELISA O157	Negative glyco-iELISA O157	Total
Positive LPS-ELISA O157	30	3*	33
Negative LPS-ELISA O157	10	8	18
Total amount of STEC-HUS patients	40	11	51

*HUS* hemolytic uremic syndrome, *LPS* lipopolysaccharide, *STEC* Shiga toxin–producing *Escherichia coli*

*Fecal diagnostics revealed O26 strain (*n* = 1), O55 strain (*n* = 1), and no further determination of serotype was performed (*n* = 1)

### Nationwide cohort of patients with TMA

The 264 serum samples of the nationwide cohort of patients with TMA sent in for STEC O157 serology were tested with both LPS-ELISA and glyco-iELISA and the outcome differed significantly (*p* < 0.0001). When the 212 samples from the patients with TMA suspicion were tested using LPS-ELISA, 48 (23%) samples were diagnosed as positive and 164 (77%) as negative (Table 4). When tested with glyco-iELISA, 60 (38%) samples of patients with TMA were confirmative for STEC O157 infection.

**Table 4:** Comparison between LPS-ELISA and glyco-iELISA in nationwide cohort of patients with thrombotic microangiopathy (TMA) of unknown etiology

Assays	Positive glyco-iELISA O157	Negative glyco-iELISA O157	Total
Total number of patients/relatives with positive LPS-ELISA O157	51	0	51
Patients	48	0	48
Relatives	3	0	3
Total number of patients/relatives with negative LPS-ELISA O157	19	194	213
Patients	12	152	164
Relatives	7	42	49
Total number of patients/relatives	70	194	264

*HUS* hemolytic uremic syndrome, *LPS* lipopolysaccharide, *STEC* Shiga toxin–producing *Escherichia coli*

Subsequently, the 52 serum samples from the relatives of patients with TMA were tested with LPS and glyco-iELISA as well (Table 4). Interestingly, 10 were positive with the glyco-iELISA indicative of STEC infection. Of these relatives, in all but one, the index patient tested positive for STEC infection as well. In this patient, mother tested positive for STEC O157; however, the patients tested negative with both glyco-iELISA, as well as LPS-ELISA.

In total, of all patients with clinical suspicion of STEC-HUS (*n* = 206, with 212 samples), 56 (27%) patients tested positive for STEC O157 infection with glyco-iELISA. Overall, using the glyco-iELISA, 19 (7.2%) patients with TMA that were previously diagnosed as negative with LPS-ELISA could be diagnosed as STEC O157 positive (*p* < 0.001).

## Discussion

Differentiation between different etiologies of HUS is highly important regarding treatment and outcome. Since aHUS is a diagnosis *per exclusionem*, proving proof of STEC infection in a “typical” STEC-HUS is essential. However, fecal diagnostics, the gold standard to diagnose STEC-HUS, have some major drawbacks, most importantly due to the natural course of disease and low inoculums. Serological diagnostics, like using anti-O157 LPS antibodies, have proven its added value to fecal diagnostics, although this diagnostic assay has a different bottleneck: potential cross-reactivity and limited sensitivity. Here, we show that the novel glyco-iELISA to detect anti-O157 antibodies is highly sensitive and specific, and its use in STEC diagnostics leads to more patients displaying positive STEC-O157 infections causing HUS. More importantly, using glyco-iELISA STEC O157-infections could be detected for a long period of time after start of the disease.

With this study, the clinical utility of the glyco-iELISA was assessed. Melli et al. were the first to publish their findings regarding this novel glyco-iELISA using bacterial-engineered glycoproteins for serotype O157, O145, and O121 [11]. In a cohort of 71 samples taken from pediatric patients (comprising both STEC-positive patients and STEC-negative patients with clinical suspicion of STEC-HUS), they showed that the glyco-iELISA was highly sensitive and specific. Furthermore, no cross-reactivity between the previous serotypes was observed, confirming our results. The same group published a second article in 2017 by Castillo et al. where they further investigated cross-reactivity between different STEC serotypes (resp. O111, O103, O45, O26, O104) and other gram-negative bacteria (salmonella, *Brucella abortus*, *Yersinia enterocolitica O*9). Again, no cross-reactivity was observed [8]. This is in contrast to LPS-ELISA, where clear cross-reactivity between different serotypes has been reported [7]. Overall, with access to the glycoproteins, glyco-iELISA is an easily implemented and performed assay with stable results.

Ideally, one would calculate sensitivity (proportion of patients with STEC-HUS in which the glyco-iELISA is positive) and specificity (proportion of patients with TMA caused by other conditions than STEC in which the glyco-iELISA remains negative) for an assay like glyco-iELISA. However, various problems arise when attempting to do so. The most prominent one has to do with the accuracy of the gold standard to diagnose STEC-HUS. Since fecal diagnostics are not sufficient to diagnose all STEC-HUS patients, no optimal gold standard is present to calculate sensitivity and specificity. Moreover, serology should not replace fecal diagnostics, but should be used in addition, to complement microbial diagnostics and broaden the time window to detect STEC. Hence, accurate estimation of sensitivity and specificity is not feasible. However, in our cohort, in contrast to LPS-iELISA, all patients with proven O157 in the feces were positive with glyco-iELISA, indicating high sensitivity (100% in our cohort). Furthermore, in patients with proven STEC infection with a non-O157 serotype, glyco-iELISA for O157 remained negative, again in contrast to LPS-ELISA for O157, indicating high specificity.

As stated previously, cross-reactivity between LPS of different gram-negative bacteria is a known problem due to the conserved lipid A part of the LPS molecule. We hypothesized that cross-reactivity, as observed in LPS-based ELISAs, can present as a false-positive test result in the O157 LPS-ELISA. This may be due to the presence of antibodies against other non-O157 STEC serotypes or even other gram-negative bacteria, which to some extent are able to bind to the lipid A part of STEC serotypes. Interestingly, using the highly specific and sensitive glyco-iELISA, we observed an increase in the detection of STEC infections, rather than a decrease due to false-positive results. Different explanations could explain this better performance. Primarily, STEC serotype O157 is still a highly prevalent serotype causing HUS in the Netherlands. Therefore, not much cross-reactivity could be found, since non-O157 serotypes causing HUS are less common. Furthermore, as shown in our single-center cohort, the glyco-iELISA is able to detect all patients with confirmed O157 serotype in the feces and remains negative in patients with a confirmed infection with other serotypes. Hence, the negative result obtained with the glyco-iELISA seemed accurate, indicating that the LPS-ELISA is probably a false-positive result due to cross-reactivity between different STEC serotypes, as was found to be the case in two of our patients. Other methods to detect serology have been reported, such as line blot immunoassay and immunoblotting, however, all use purified LPS to detect antibodies; hence, potential cross-reactivity remains present [7].

Interestingly, of the 52 samples of relatives without clinical HUS features, 10 (19.2%) had antibodies detected with the glyco-iELISA, indicating STEC O157 transmission person-to-person or intake of the same contaminated food. In all except one, the index patient tested positive for STEC infection. Although we have no clinical information about relatives in our study, we could show that family members of STEC-HUS patients with no or mild signs of gastrointestinal infection can develop antibodies against O157. These results are in line with Ludwig et al. who reported that 17% of the household contacts (symptomatic as well as asymptomatic) of STEC-HUS patients had LPS IgM antibodies against STEC serotype O157 [20]. The exact rate of IgM antibodies against STEC serotypes in the healthy population is still unknown. However, to exclude potential false-positive results, one could consider only testing for the presence of IgM and not IgG, since IgG can be present for years after infection. Yet, we would recommend testing family members of patients with STEC (with fecal diagnostics and serology), especially in patients who tested negative for STEC infection. By providing proof of STEC infection in household contact, the diagnosis of STEC-HUS in the index patient despite negative diagnostics becomes more likely.

We found IgM antibodies against STEC O157 up to 55 days after onset of the disease. These results are in line with previous reported kinetics of IgM (LPS-based assay) against STEC by Chart et al. [21]. Hence, in contrast to fecal diagnostics in which the isolation rate declines quickly after the initial symptoms (within 1 week), serology (both LPS- and glyco-iELISA) broadens the time window to diagnose STEC infections. Furthermore, when serum is collected too early in the course of the disease, serology could be negative due to as yet incomplete seroconversion, as was the case in two of our patients. In case of a negative serology result tested in serum collected within 7 days after disease onset, the advice would be to collect and test serum again after 7 days for a re-evaluation. As described previously, the added value of serology increases even more 7 days or more after the start of the symptoms [6].

Limitations of this study are the retrospective nature and the lack of clinical data of the national TMA cohort. Serological detection of STEC infection by detection of anti-O157 antibodies in serum is advised in the national guideline of diagnostic workup for TMA at presentation. Presumably, some patients in the national cohort had a different diagnosis that not only comprised STEC-HUS, but also aHUS or other causes of TMA. Concerning the single-center cohort of pediatric patients, STEC infection could not be detected in seven patients with clinical suspicion of HUS. Yet, aHUS as a diagnosis is highly unlikely regarding the clinical presentation with bloody diarrhea in all seven patients. Although aHUS can present in 30% of cases with gastrointestinal infection, bloody diarrhea is seldom reported in aHUS. Also, follow-up data showed no disease recurrence, making aHUS highly unlikely in this single-center cohort. In three patients, genetic analysis was performed and showed no pathogenic mutations in complement genes associated with aHUS. Furthermore, in most patients, serology was only tested at one time point. Seroconversion takes 3–5 days; hence, patients who were seen early in the course of disease could be false negative. Furthermore, we focused on the still most prevalent STEC serotype causing HUS in our country, O157; however, nowadays, non-O157 serotypes are increasingly detected as a cause of HUS. The relatively high number of patients with STEC O157 infection in our cohort could be explained by the substantial number of STEC-HUS patients who were included from the late 1990s, when serotype O157 was, as it still is, the main serotype to cause STEC-HUS. Nowadays, non-O157 serotypes causing HUS are increasingly detected, partly explained due to new and improved diagnostic assays. In this study, non-O157 STEC serotypes were not detected [2, 4]. It would be very worthwhile in the near future to examine the 14% of clinical STEC-HUS patients who were negative in fecal and serological diagnostics for other STEC serotypes with glyco-iELISA. Hence, future plans are to expand glyco-iELISA to detect multiple serotypes. Melli et al. have already described the use of glyco-iELISA for STEC serotypes O145 and O121, with comparable results regarding absence of cross-reactivity and sensitivity of the assays [11]. Furthermore, it is highly important to differentiate between STEC-HUS and aHUS as soon as possible, to start appropriate treatment. Since the current glyco-iELISA takes at least 24 h to perform, future studies should focus on improving this assay for bedside use. For example, by using lateral flow technology, one could develop a point of care test for patients presenting with TMA.

In conclusion, serological assays for STEC O-antigens have a place in the diagnostic workup plan of patients with TMA. Moreover, since aHUS is a diagnosis *per exclusionem*, it is highly important to diagnose STEC-HUS. Therefore, we advocate always combining fecal diagnostics together with serological diagnostics to achieve optimal diagnostics and prevent unnecessary use of the highly expensive orphan drug eculizumab. The optimal assay to determine serological antibodies against STEC serotype O157 is the glyco-iELISA.
